# Data Collection for Mobile Group Consumption: An Asynchronous Distributed Approach †

**DOI:** 10.3390/s16040482

**Published:** 2016-04-06

**Authors:** Weiping Zhu, Weiran Chen, Zhejie Hu, Zuoyou Li, Yue Liang, Jiaojiao Chen

**Affiliations:** 1 International School of Software, Wuhan University, Wuhan 430079, China; zjhu@whu.edu.cn (Z.H.); zuoyouli@whu.edu.cn (Z.L.); lymoondrink@whu.edu.cn (Y.L.); jiaojiaochen@whu.edu.cn (J.C.); 2 State Key Lab. for Novel Software Technology, Nanjing University, Nanjing 210046, China; 3 Economics and Management School, Wuhan University, Wuhan 430079, China; wrchenwhu@163.com

**Keywords:** asynchronous, distributed, data collection, mobile group consumption

## Abstract

Mobile group consumption refers to consumption by a group of people, such as a couple, a family, colleagues and friends, based on mobile communications. It differs from consumption only involving individuals, because of the complex relations among group members. Existing data collection systems for mobile group consumption are centralized, which has the disadvantages of being a performance bottleneck, having single-point failure and increasing business and security risks. Moreover, these data collection systems are based on a synchronized clock, which is often unrealistic because of hardware constraints, privacy concerns or synchronization cost. In this paper, we propose the first asynchronous distributed approach to collecting data generated by mobile group consumption. We formally built a system model thereof based on asynchronous distributed communication. We then designed a simulation system for the model for which we propose a three-layer solution framework. After that, we describe how to detect the causality relation of two/three gathering events that happened in the system based on the collected data. Various definitions of causality relations based on asynchronous distributed communication are supported. Extensive simulation results show that the proposed approach is effective for data collection relating to mobile group consumption.

## 1. Introduction

Group consumption refers to consumption by a group of people; for example, a couple having dinner at a restaurant, two friends watching a film at the cinema or a girl, accompanied by two of her colleagues, going shopping to buy a skirt. The people in the group usually have certain relations, and the opinion of any person in the group may affect the choice of goods or services being consumed. This kind of consumption differs from consumption that only involves individuals, because the consumption decision results from interaction and negotiation among group members. Group consumption is not uncommon in our daily lives. In fact, when people are in public places, 70% of their time is spent in the company of other persons [1]. In many cases, they participate in group consumption.

**Figure 1:**
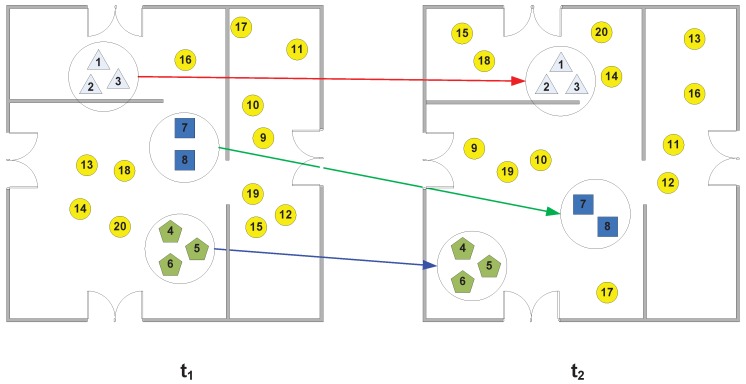
An illustration of mobile group consumption. Several people wander around a shopping mall with different regions. Each person is denoted by a node, and those belonging to groups have the same shape, except for the circle. The circle nodes denote the people who do not belong to groups (*i.e*., individuals). The picture on the left is a snapshot of people at timestamp $t_{1}$, and the picture on the right is a snapshot of people at timestamp $t_{2}$ after $t_{1}$.

In recent years, a growing number of people have been using mobile phones to scan, search and receive information from retailers and to order various services and goods. According to a survey by the InMobi Insights Team, about 46% of people have made purchases using their mobile devices, and 80% of people plan to do so in the next 12 months [2]. We refer to group consumption based on mobile devices as *mobile group consumption*. An example thereof can be seen in Figure 1. Having observed this trend, many companies increasingly invest in marketing efforts to promote consumption based on mobile devices. In 2014, 32.7 billion U.S. dollars were spent globally on mobile advertisements, which is about one fourth of the total network advertisements [3]. However, current mobile advertising does not sufficiently consider the role of group characteristics in consumption.

The analysis of mobile group consumption first requires an effective data collection system to be built. Typical data that need to be collected include the trajectories and actions of group members for a specified time interval. Various technologies can be used to achieve this purpose. For example, WiFi signals transmitted from access points to consumers’ mobile phones can be collected. The RFID tags attached to mobile phones could also be read for analysis.

Existing data collection systems [4,5,6] that can be used for mobile group consumption are based on centralized processing. A central server is needed to gather information from consumers. These systems encounter the following challenges: (1) in many scenarios, it is difficult to find a server that is appropriately powerful; (2) even if there is a suitable server available, it presents a single point of failure, a computational bottleneck, especially in the scenarios including a large number of shops and consumers and performing complex data processing after data collection; (3) the users may not be willing to share their data with a central server due to security or business concerns. Moreover, existing work assumes the existence of a synchronized clock, which incurs high overhead when there is a large number of constantly-moving consumers. Sometimes, synchronization is even impossible because of device hardware constraints or people’s privacy concerns. A new approach is highly demanded to solve the aforementioned problems.

In this paper, we present an asynchronous distributed data collection approach designed to analyze mobile group consumption. Our approach does not rely on a central server or synchronized clock; instead, it relies on distributed and asynchronous message communications. We first built the system model for asynchronous distributed data collection. Based thereon, we designed a simulation system. Then, we proposed a three-layer solution for generic distributed processing, including the processing for local sub-regions, consecutive sub-regions and multiple sub-regions. We subsequently developed our proposed approach to detect the causality of two specified marketing activities of group consumption. Extensive simulations were carried out to evaluate our approach. The results showed that the approach can effectively support data collection of mobile group consumption. In summary, this paper makes the following contributions:

- We build the system model of mobile group consumption based on asynchronous message passing, *i.e*., it is not based on a centralized server or synchronized clock. We also describe the simulation system developed based on this model.
- We propose a three-layer mechanism to collect data for mobile group consumption in an asynchronous distributed way. The data collection is firstly handled locally and then coordinated in convective or more regions if the data collection spans a wide area.
- We conduct extensive simulations to validate the proposed approach. The results show that the proposed algorithm is quite effective.

The remainder of the paper is organized as follows: Section 2 presents the system model of asynchronous distributed data collection that we built for mobile group consumption. Section 3 explains the simulation system based thereon. The detailed data collection solution is described in Section 4. The simulation results are reported in Section 5. Section 6 reviews related work, and Section 7 concludes the paper.

This paper is based on our previous conference paper [7] published in Proceedings of the International Conference on Identification, Information & Knowledge in the Internet of Things (IIKI) 2015. In this version, we add the design of our simulation system, extend the approach to support more complex temporal relations for causality detection, support the message passing with arbitrary speed and also provide more analytical details and simulation results.

## 2. System Model

We assume that mobile group consumption happens in a place (e.g., a shopping mall) where shops broadcast promotion information from time to time. Groups of shoppers wander around and may be attracted by one or more promotions. Each group includes one or more members. Each promotion is assumed to last for a certain period of time. People can only receive information about promotions from a shop if they are located in a specified area of the shop, referred to as the *affecting area*. If *N* persons are gathered in a shop during its promotion time, we refer to this as a *gathering event*. Each gathering event exists in a time interval, which is delimited by the start time of the gathering event (the number of gathered people exceeds *N* for the first time) and the end time of the gathering event (the number of gathered people is less than *N* for the first time). There is a sensing system to collect and analyze the behavior of customers during mobile group consumption. Our system does not have a central server.

**Figure 2:**
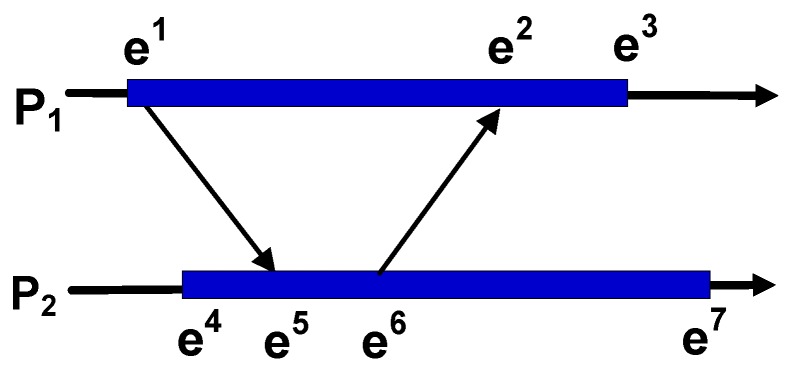
Temporal relations based on different kinds of clocks. $P_{1}$ and $P_{2}$ are devices, each of which is represented by a time axis oriented from the left to right. $e^{1},e^{2}...$ are events that happened in devices at different timestamps. An arrow from one event to another represents a message transfer. According to the physical clock, the sequence of events can be directly determined by their timestamps (e.g., $e_{3}$ is before $e_{7}$). According to the logical clock, the sequence of events is determined by message passing (e.g., $e_{1}$ is before $e_{5}$) or local timestamps (e.g., $e_{1}$ is before $e_{2}$).

Temporal relations in this system can either be based on a *physical clock* or a *logical clock*. For the physical clock, all of the clocks in different devices are synchronized in appropriate ways. The temporal sequence of events can be determined by measuring the timestamps of the synchronized clocks. The logical clock does not depend on a synchronized clock in the system. In this case, the sequence of events can only be determined by the exchange of messages among devices or timestamps in the same device [8]. Figure 2 shows the differences between temporal relations based on the physical clock and the logical clock, where $P_{1}$ and $P_{2}$ are devices and $e^{1},e^{2}...$ are events that happened in the corresponding devices. According to the physical clock, the sequence of $e^{3}$ and $e^{7}$ can be directly determined by their timestamps. However, according to the logical clock, except for the sequence of events that, in the same devices (e.g., $e^{1}$ and $e^{2}$), can be determined by their timestamps, the sequence of events can only be determined by message passing. Since there is a message from $e^{1}$ of $P_{1}$ to $e^{5}$ of $P_{2}$, it can be concluded that $e^{1}$ happens before $e^{5}$. Similarly, $e^{6}$ happens before $e^{2}$. If message passing among devices has a finite and arbitrary time delay, we refer to it as *asynchronous communication*.

Let us define the temporal relations under asynchronous communications more formally. Suppose that there are *n* devices $P_{i}$ (i=1...n) and that each device $P_{i}$ records its alternate states and events $s_{i}^{0},e_{i}^{0},s_{i}^{1},e_{i}^{1},...,e_{i}^{j},s_{i}^{j}$ [9], where $s_{i}^{k}$ denotes the *k*-th state and $e_{i}^{k}$ denotes the *k*-th event that changes the local state from $s_{i}^{k-1}$ to $s_{i}^{k}$, at the device $P_{i}$.

We refer to this state as $s_{a}$ *happens before* state $s_{b}$, denoted by $s_{a}\to s_{b}$, if:

(1) $s_{a}$ is a state before $s_{b}$ in the same device;
(2) the event immediately after state $s_{a}$ sends a message and the event immediately before state $s_{b}$ receives that message;
(3) there is a state $s_{c}$, such that $s_{a}\to s_{c}$ and $s_{c}\to s_{b}$ [10].

If $s_{a}$ does not happen before $s_{b}$ and $s_{b}$ does not happen before $s_{a}$, we say that $s_{a}$ is *concurrent with* $s_{b}$, denoted by $s_{a}\;\left|\right|\;s_{b}$.

## 3. Simulation System

**Figure 3:**
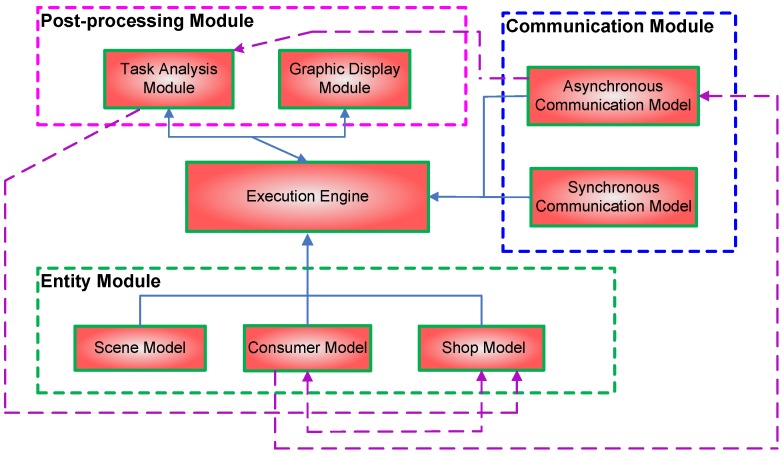
The architecture of the simulation system for mobile group consumption. The solid arrows represent data flows. The dashed arrows represent the influence between two models.

In order to investigate the problem efficiently, we developed a simulation system named the Group Consumption Simulation system (GCS). The top-level architecture of this system can be seen in Figure 3. This system includes four modules: the execution engine, the entity module, the communication module and the post-processing module.

At the core of the system, the execution engine controls the whole simulation process and triggers the execution of other modules. The execution engine configures the simulation environment based on the entity module, performs the simulation based on the communication module and finally conducts the analysis and displays the graphics based on the post-processing module.

The entity module includes three major models: the scene model, the consumer model and the shop model. The scene model defines the area of the shopping mall, the layout of shops in the mall, the composition of each group and the initial location and moving direction of each person. The consumer model defines the role of each member in a group (e.g., group leader, normal member), the actions in reaction to a promotion and the motion patterns in the shopping mall in terms of groups and individuals. It is noted that the motion patterns in open spaces differ from those after being attracted to a shop. Finally, the shop model defines the promotion time, promotion duration and promotion frequency of each shop.

The communication module includes both an asynchronous communication model and a synchronous communication model, which are based on message passing and the global clock, respectively. The asynchronous communication model follows our specification in Section 2, which is the major concern of this paper. The synchronous communication model assumes that each entity has a synchronized clock and can be used as the ground truth for comparison with message passing.

The post-processing module includes the task analysis module and the graphic display module. The task analysis module allows users to define their tasks of interest; for example, the causality of different promotions, the impact of a promotion in an area, *etc*. The graphic display module is used to display the results of the analysis graphically.

It is noted that some models may affect each other. These influences are indicated using dashed lines in Figure 3. Consumers’ motions may affect the promotion strategy of a shop, and the promotion strategy of a shop may also affect consumers’ motions. Therefore, there are bidirectional dashed lines between the consumer model and the shop model. The shop model may also be affected by the results of the task analysis module. In the case of the asynchronous communication model, the parameters of message passing may be affected by consumers’ motions, on the one hand, and affect the results of the task analysis module, on the other. The impact of different models is left for future work and is not implemented in the current system.

## 4. Data Collection Approach

In this section, we present the algorithms that are used to collect data for group consumption in an asynchronous distributed way.

### 4.1. Distributed Data Collection

**Figure 4:**
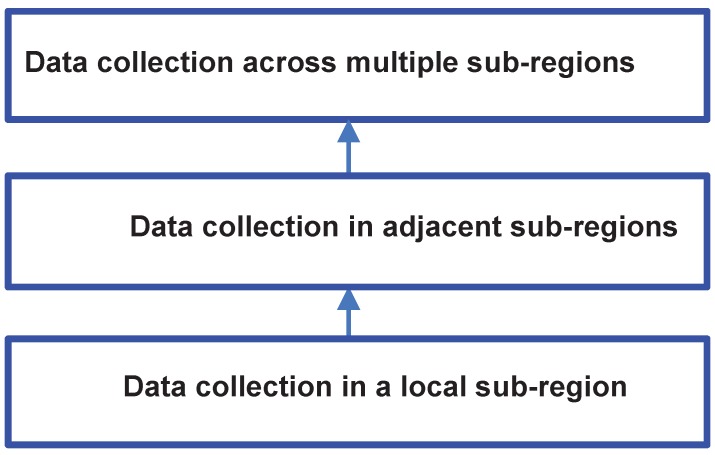
A three-layer distributed data collection framework.

We propose a three-layer solution for distributed data collection of mobile group consumption. The whole target region is split into several sub-regions. As shown in Figure 4, the first layer processes data collection in a sub-region; the second layer processes that in adjacent sub-regions; and the third layer processes that across multiple sub-regions or even across the entire region. Most of the data collection requirements can be processed locally in the first layer. However, local processing is not necessarily optimal or correct. For example, users may need to collect data in a 10-m${}^{2}$ area centered at *A*. Local processing is acceptable when *A* is located within a sub-region, because the considered area is included in the sub-region, whereas it may be incorrect when *A* is near the boundaries of a sub-region, because the considered area may span two or more adjacent sub-regions. The second layer can be used to coordinate data from adjacent regions. Other data collection requirements that involve multiple sub-regions can be coordinated in the third layer.

### 4.2. Asynchronous Distributed Data Collection

We further propose the Asynchronous Distributed Data Collection Approach (ADDC), which can determine the order of different events based on asynchronous communications. We do not assume a central server or synchronized clock in the system. The order in which all events occur is determined by messages exchanged among devices. More specifically, the order is based on vector clocks [11] maintained in the system.

We explain the approach using a typical example from mobile group consumption, the analysis of causality relations. It is briefly described as follows: A promotion offered by a shop is regarded to be effective if a gathering event happens. Two gathering events are considered to have a causality relation if they have temporal sequences (e.g., one gathering event happens before another). In this section, we identify all such pairs of gathering events.

**Figure 5:**
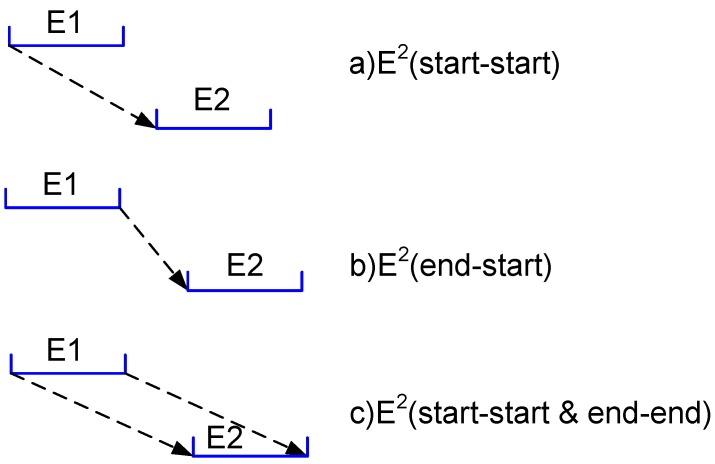
Causality detection based on three different definitions. Each event is presented by a segment. The message passing is presented by a dashed arrow from the sender to the receiver.

In a classical system with synchronized clocks, a causality relation can be detected based on the value of the clocks directly, whereas in an asynchronous distributed system, such clocks are not available. Therefore, we need to utilize message passing to detect the causality relation, which can be based on different definitions. We describe the most common ones in Figure 5. In the first case, the causality is based on the start time of the first gathering event and the start time of the second gathering event. We refer to it as $E^{2}\left(start-start\right)$, where 2 refers to the two events considered and *start*-*start* refers to the delimitation time of two gathering events. In the second case, the causality is based on the end time of the first gathering event and the start time of the second gathering event. We refer to it as $E^{2}\left(end-start\right)$. In the third case, the situation is more complex. The causality is based on the fact that the start time of the first gathering event happens before the start time of the second gathering event, and the end time of the first gathering event happens before the end time of the second gathering event. We refer to it as $E^{2}\left(start-start\;\&\;end-end\right)$. The dashed lines in Figure 5 denote the message passing between the two events, hence their “happen before” relation. Our algorithm can support these three definitions.

Our approach specifies the actions of two kinds of entities in the system: consumers and shops. The actions of consumers are specified in Algorithm 1. The actions of shops are specified in Algorithms 2 and 3. Subsequently, Algorithm 4 specifies the coordination of the processing occurring in the sub-regions to handle a user’s detection requirement. The notations used in the algorithms are summarized in Table 1. We illustrate the functions performed by each of the algorithms as follows.

Algorithm 1 specifies the actions that are performed by consumers. The first action triggers the detection of events related to causality relations. When consumers are in the affecting area of a shop and the shop is broadcasting promotion information at that moment, the consumers can decide, by themselves or by consulting within the group, whether to enter the shop to obtain more details (Line 1). If the consumers decide to accept the promotion and enter the shop (Line 2), their smart mobile devices update their locations to the shop (Line 3) and send messages to the shop they have entered to notify them of this action (Line 4). Similarly, when the consumers leave the scope of the shop (Line 6), their corresponding smart mobile devices update the locations and notify the shop (Lines 7,8). The enter message and exit message can trigger the shop to detect the start time and the end time of gathering events. The other action performed by consumers is message relay, which is to forward the start message or end message of gathering events from the initiator to others to build the “happen before” relations. The order in which different events occur is based on vector clocks used in the asynchronous system. Thus, the vector clocks in the devices are firstly updated (Lines 10,11) before corresponding messages are sent to the neighboring shops or people (Line 12).

**Algorithm 1:** Asynchronous Distributed Data Collection Approach (Consumer)
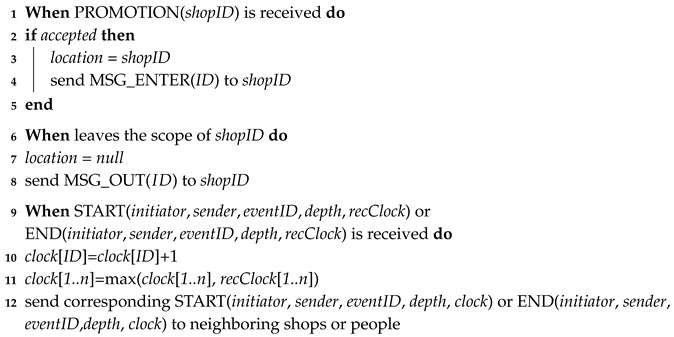


Algorithms 2 and 3 specify the actions performed by shops (we split the process into two algorithms because of page size constraints). They include the detection of the start time and end time of gathering events, the detection of causality relations based on different definitions and the building of a routing tree whose root can finally store detection results.

We illustrate the details as follows. When an enter message is received from a consumer (Line 1), the recorded number of consumers in the shop and the detailed IDs of the consumers are updated firstly (Lines 2,3). If the recorded number of consumers is greater than the threshold *gatherThrehold*, a new gathering event is detected (Line 4). The local clock is increased by one (Line 5), and the function *increaseID* is invoked to generate the event ID (Line 6). The new event ID is a string including the ID of the shop and also a local event sequence ID (e.g., shop1_event6). It is used to distinguish different events generated by shops. After that, a relay message is sent to people within and around the shop (Line 7). These people move around and/or send the information to their neighbors; hence, the “happen before” relation is built.

When a new gathering event is found, the algorithm further detects the causality relation. We use SS to denote $E^{2}$(start−start), ES to denote $E^{2}$(end−start) and SSEE to denote $E^{2}\left(start-start\;\&\;end-end\right)$. If the current detection is based on SS, the start time of a previous gathering event (recorded in *oriStart*[*preEventID*]) leads to a successful detection (Lines 9,10). Similarly, the detection based on ES can be seen in Lines 11,12. If the detection is based on SSEE, only the first part can be complete; the start time of the first gathering event happens before the start time of the second gathering event. The result is recorded for further processing (Lines 13,14).

Similarly, when an exit message of a consumer is received (Line 18), the recorded number of consumers in the shop and the detailed IDs of the consumers are updated (Lines 19–20). If the recorded number of consumers is less than *gatherThrehold*, the end time of the existing gathering event is detected (Line 21). The vector clock is updated locally (Line 22), and a message is sent to people within and around the shop (Line 23). The causality detection based on SSEE can be completed here by checking the second part; the end time of the first gathering event happens before the end time of the second gathering event. If it holds, the whole causality relation is detected, and a successful message is sent out (Lines 24–27).

**Algorithm 2:** Asynchronous Distributed Data Collection Approach (Shop).
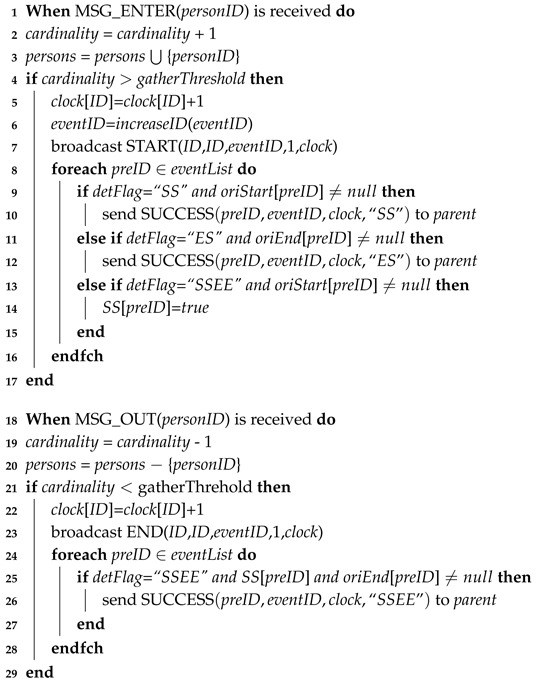


When the messages START and END are received via forwarding by consumers, the shop records the initiator of the message and the event ID (Lines 32,33,44,45). Since the consumers are moving all of the time, we build a routing tree based on fixed shops for message exchange. The detection results of a causality relation are transmitted along the routing tree. This routing tree is built as follows: when a relay message is received for the first time, the shop sets its parent in the routing tree as the sending node (Lines 34,35,46,47). The depth of the node in the tree is also updated (Lines 36,48). In order to achieve a routing tree of small depth, we design a depth-adaptive routing tree. When the depth of the routing tree exceeds a threshold *depthThreshold*, the current shop automatically becomes the root, and a reverse message is sent to its previous ancestors (Lines 37–39, 49–51). When the reverse information is received by a shop, it updates its parent to the sender of the message and further forwards the message to its antecessors (Lines 54–57). The clock operations when receiving the messages START and END are similar to those in Lines 10,11 of Algorithm 1 and are omitted here.

**Algorithm 3:** Asynchronous Distributed Data Collection Approach (shop) (cont.).
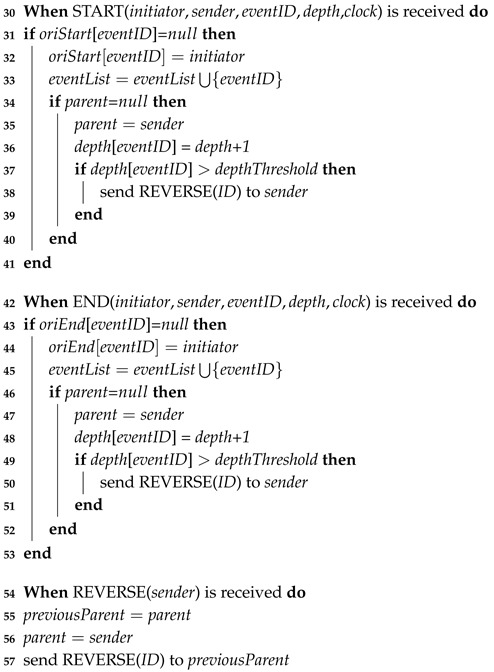


Finally, Algorithm 4 illustrates the coordination of the processing of the users’ detection requirements by sub-regions. Usually, a user’s detection requirement involves several sub-regions. We first calculate such sub-regions and include them in a set *S* (Line 2). The event to be detected and the calculated sub-regions are sent to the children nodes of the requesting node in the routing tree (Line 3). The message is forwarded in the tree, and each forwarding node waits for the results of its children (Lines 8–10). When a node belonging to a needed sub-region is detected for the first time, the node changes its flag *coordinator* (with regards to the current event) to true, which marks it the coordinator of its sub-region (Lines 5–7). If a leaf node in the routing tree is detected to belong to *S*, related events and the node ID are recorded (Lines 12,13) and sent to the leaf node’s parent (Line 14). Once the results of all children have been received, the node forwards the results to its parent (Line 23), after collecting local events and the node ID if it is determined as being in *S* (Lines 18–21). If the node is a coordinator (*coordinator*[*E*]=*true*), it compares the accumulated area to *S*. When more detection is needed, the coordinator forwards the result to its neighboring sub-regions to generate the final results (Lines 24–26).

**Algorithm 4:** Multiple sub-Region Coordination Algorithm for Causality Analysis.
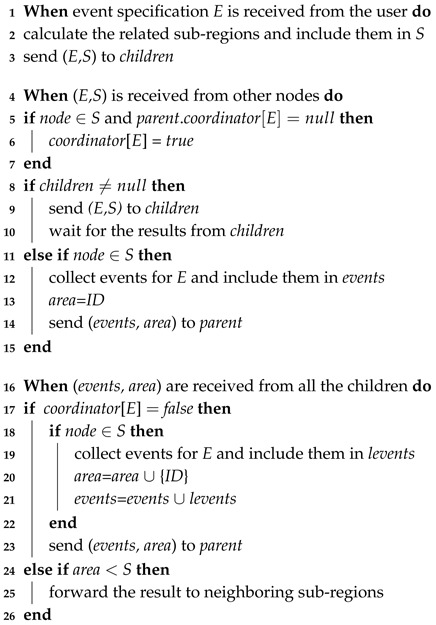


### 4.3. Complexity of the Algorithms

Let us discuss the complexity of the algorithms. Suppose a shop broadcasts promotion information, and several persons are attracted to the shop. Each such person needs to send a MSG_ENTER message and a MSG_EXIT message to the shop. During the promotion, each person forwards one or more START and END messages to others. The number of messages forwarded depends on the relative location of the person to others. When the person moves to other shops, he or she may forward the START and END messages to those shops. Considering that there are multiple shops in a shopping mall (so multiple events), each person may forward more START and END messages received from other shops, where the number of messages depends on the frequency of these shops’ promotions and the relative locations of the persons to these shops. During the promotion, the shop broadcasts a START message and an END message to the people within or around it. When a shop receives the START or END message from others, it builds a routing tree first and detects causality relations. The time complexity of routing tree building and causality detection is O(*n*), where *n* is the number of shops. Several REVERSE messages may also be sent to optimize the routing tree. Each REVERSE message affects all of the shops from the current shop to the root. This needs O(*d*) messages, where *d* is the depth of the routing tree. Besides the process above, we also need to transmit the event specification from the root to specific shops and to forward the results to neighboring sub-regions. The former needs O(*d*) messages, and the number of messages of the latter depends on the events to be detected. The energy consumption of shops in this problem is not a concern, and that of consumers can be calculated based on the number of messages sent out.

It is noted that the exact computational complexity of the algorithms depends on many factors, including the frequency of shops’ promotions, the number and layout of shops in the shopping mall, the number of people and their groups, the motion of people and the relations of people in a group. It is difficult to analyze this in a general case. The improvement for the complexity of the algorithms with multiple events and groups is left as future work.

### 4.4. Discussion

In this section, we discuss several issues related to asynchronous distributed data collection.

First, Algorithm 2 works under a moderate speed of message passing. We use an example to explain this. Suppose that Gathering Event A of Shop X happens before Gathering Event B of Shop Y in the real world, and we want to detect their causality relation based on asynchronous communications. The START message of A needs to reach Y before B happens, if the detection is based on SS, then the causality relation can be detected. Since the message passing among shops and consumers is uncertain under asynchronous communications, this assumption cannot be guaranteed in some cases. This means that Y may receive the START message of A (say M) after the START message of B (say N), in spite of M happening before N. We addressed this problem by revising the algorithm to invoke the detection logic (Lines 9–15, 25–27) once each message is received. The event order is determined based on the vector clocks of *preID* and the current event, besides *oriStart*[*preID*] ≠null.

The second concern is complex temporal relations based on asynchronous communications. ADDC detects causality relations according to the temporal relations based on time points. More complex temporal relations can be based on time intervals. There are 29 kinds of relations between two time intervals under the dense time mode and 40 kinds of relations under the non-dense time mode [8,12]. Moreover, the temporal relations under asynchronous communications often cannot be determined with certainty. This problem can be overcome by using occurrence probability [13] to specify the probability that a relation holds.

Finally, it is noted that the causality analysis in this paper is based on logical temporal relations under asynchronous communications. This is a little different from the temporal relations based on physical time that can specify a time duration between two gathering events. Such a quantity measurement is difficult to perform in the logical time, because a synchronized clock is not available. If the exchange of messages among devices is sufficiently frequent, we can use the number of messages from one event to another event to estimate the time duration between them. However, the effectiveness of such a solution would vary depending on the message passing scenario.

## 5. Performance Evaluation

We conducted simulations to validate the effectiveness of our proposed ADDC approach by comparing the results obtained with ADDC with those measured using the physical clock. The detection of causality relations among two/three gathering events is used as the comparison example. The number of event patterns satisfying the causality relation among two and three gathering events is denoted by $E_{T}^{2}$ and $E_{T}^{3}$, respectively. For detection under the physical clock, we specify the duration between two gathering events *T* to be 500 s. For detection using ADDC, we need two gathering events to have the “happen before” relation (*T* is of no use in this detection). The time duration of $E_{T}^{2}$ is measured from the start time of the first gathering event to the start time of the second gathering event, and that of $E_{T}^{3}$ is measured from the end time of the first gathering event to the start time of the second gathering event.

### 5.1. Simulation Setup

**Figure 6:**
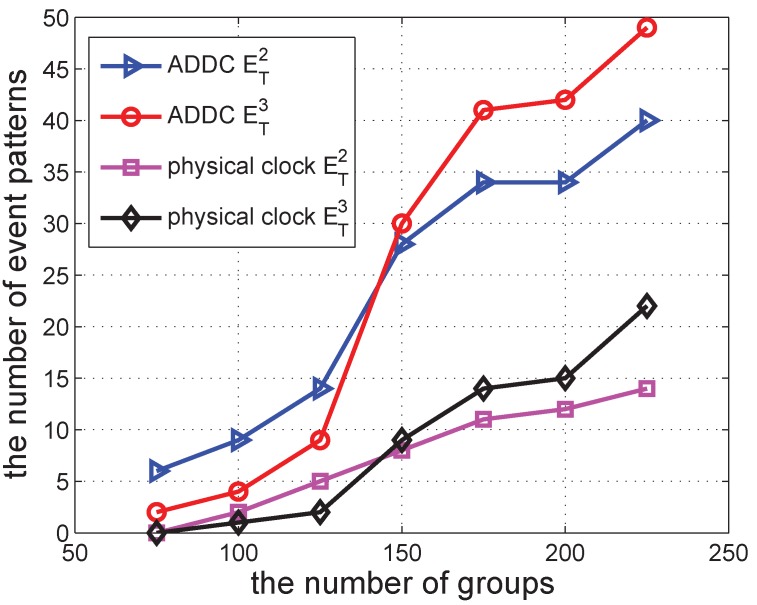
The number of event patterns *vs.* the number of groups.

**Figure 7:**
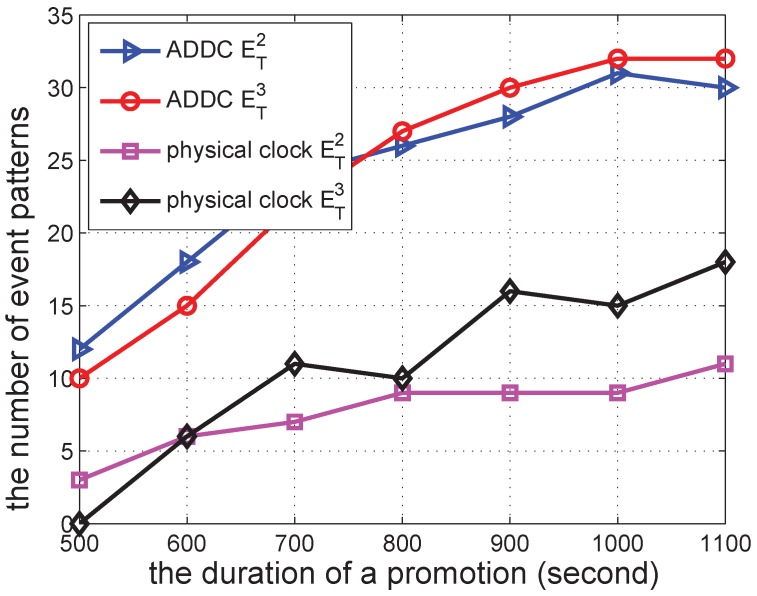
The number of event patterns *vs.* the duration of a promotion.

**Figure 8:**
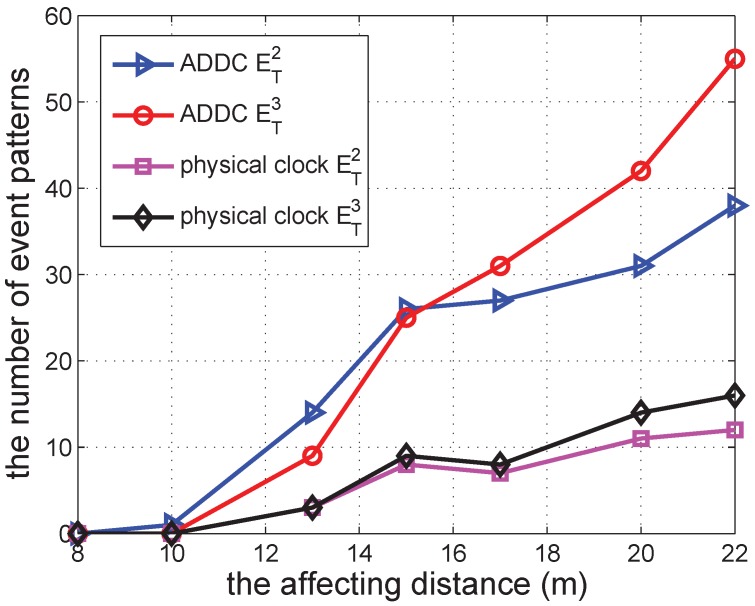
The number of event patterns *vs.* affecting distance.

**Figure 9:**
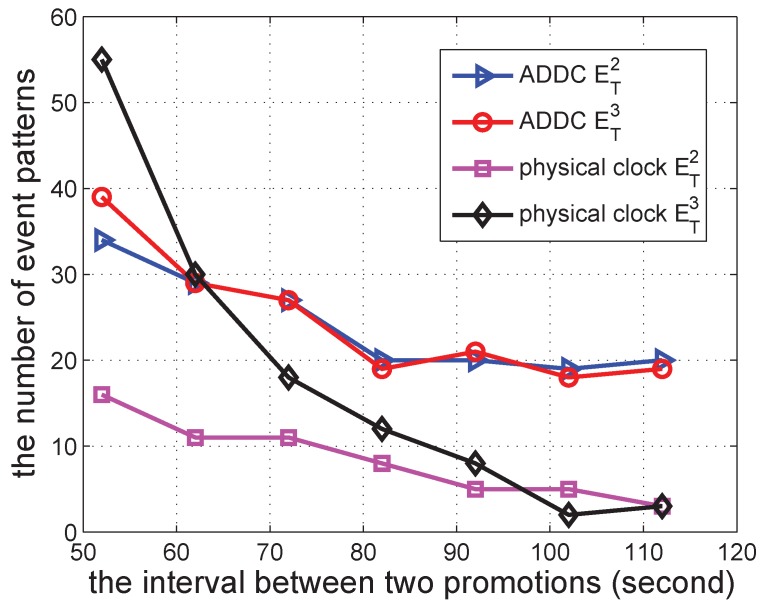
The number of event patterns *vs.* the interval between two promotions.

**Figure 10:**
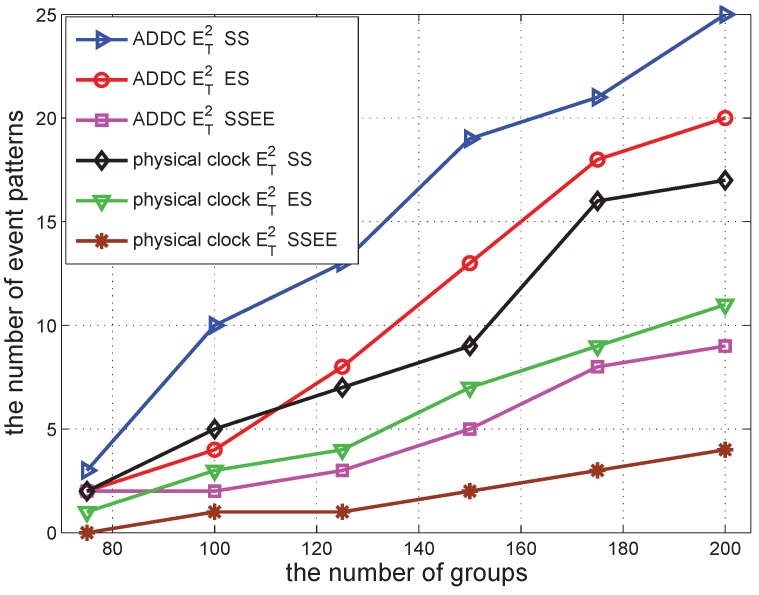
The number of event patterns *vs.* the number of groups.

**Figure 11:**
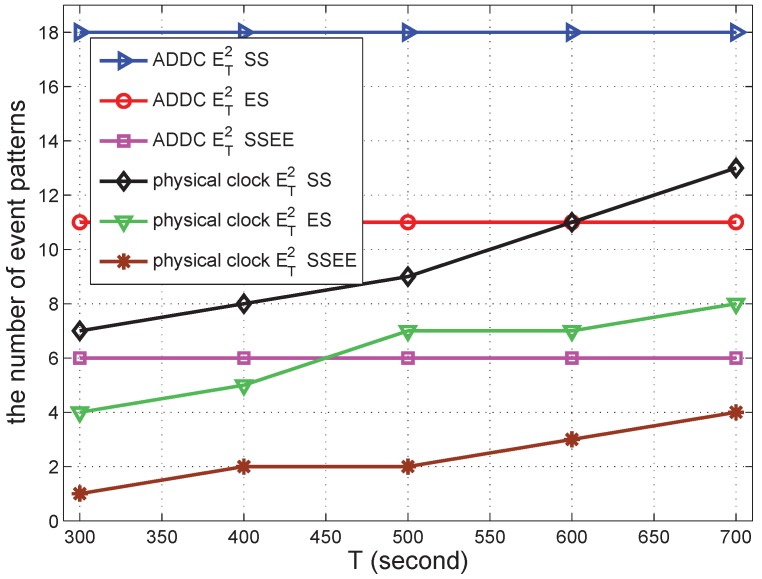
The number of event patterns *vs.* T.

**Figure 12:**
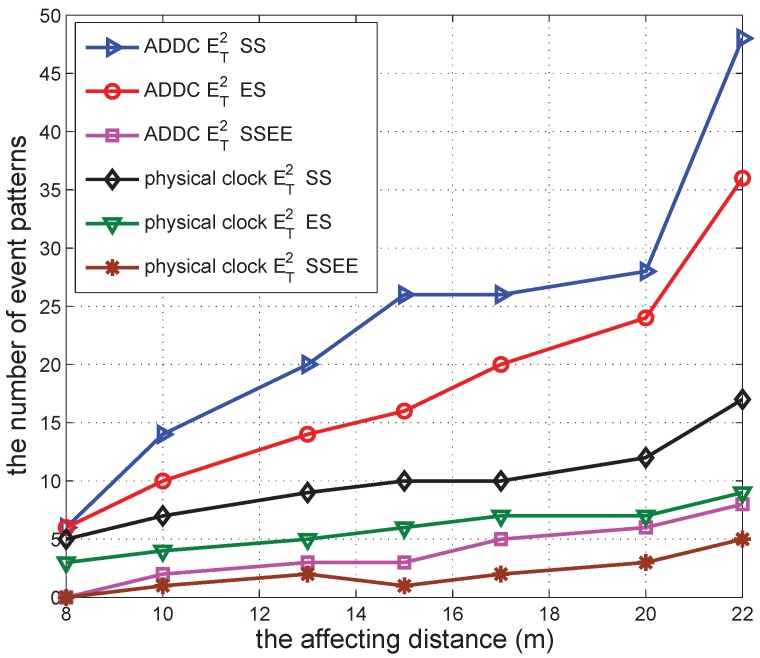
The number of event patterns *vs.* affecting distance.

**Figure 13:**
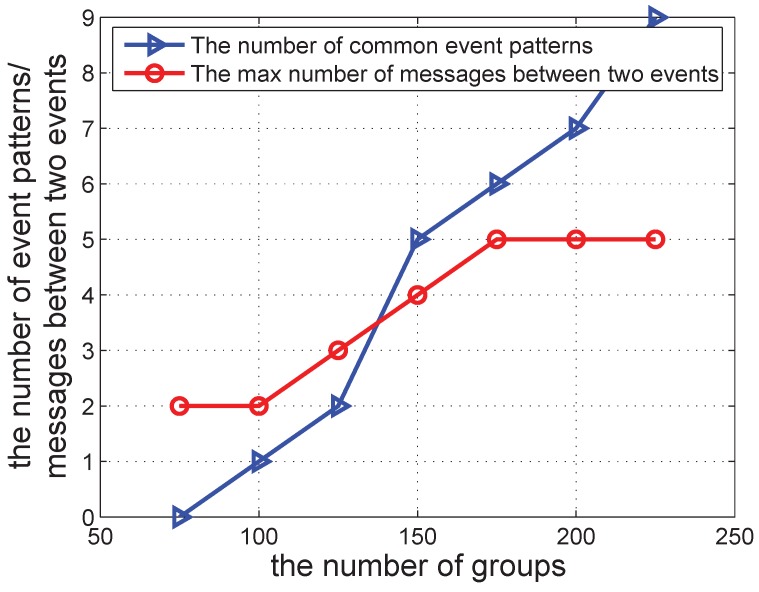
The number of common event patterns/the max number of messages between two events *vs.* the number of groups.

**Figure 14:**
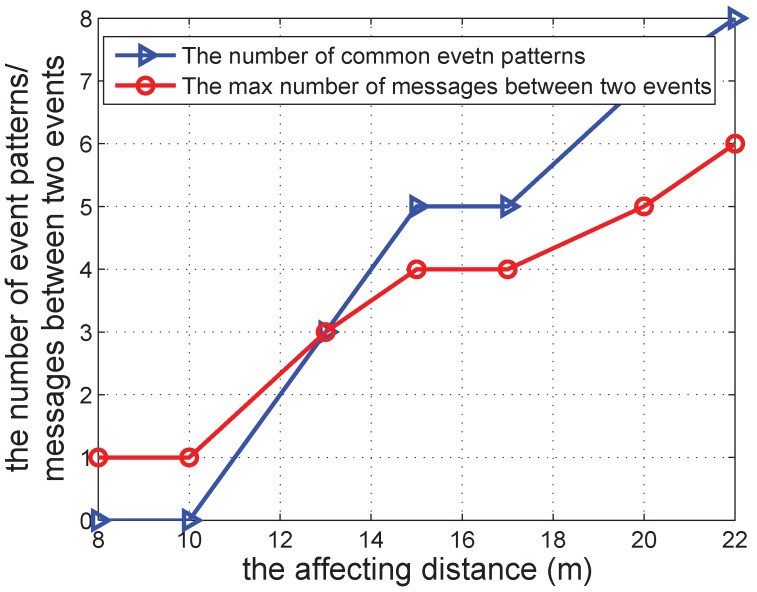
The number of common event patterns/the max number of messages between two events *vs.* the affecting distance.

In the following several sub-sections, we simulate a shopping mall of 10 m × 10 m containing 12 shops and with several groups of people wandering around. In each second, each shop begins a promotion with a probability $p_{o}=0.00307$ to attract people, and this lasts for a duration *s*. The promotion can attract people from within a distance of d<15 m. When there is a promotion, the group can be attracted with a specified probability of $p_{m}=0.49$. We vary different parameters in the simulations to check the performance of our approach. The simulation was repeated 30 times to obtain each data point in Figure 6, Figure 7, Figure 8, Figure 9, Figure 10, Figure 11, Figure 12, Figure 13 and Figure 14.

### 5.2. The Number of Event Patterns Detected

We first vary the number of groups in the shopping mall to check the detected event pattern of $E_{T}^{2}$ and $E_{T}^{3}$. The result is shown in Figure 6. It shows that the number of $E_{T}^{2}$ and $E_{T}^{3}$ increases when the number of groups increases. When the number of groups is 75, the number of $E_{T}^{2}$ and $E_{T}^{3}$ detected by both ADDC and the physical clock is small, less than seven. The number of $E_{T}^{2}$ detected by ADDC is increased to around 40 when the number of groups reaches 225. The number of $E_{T}^{3}$ detected by ADDC is also increased when the number of groups increases, but at a higher rate. Its number is smaller than that of $E_{T}^{2}$ when there are fewer than 150 groups. This is because the constraint of $E_{T}^{3}$ is more strict than that of $E_{T}^{2}$. Moreover, its number becomes larger than that of $E_{T}^{2}$; the number of groups exceeds 150. This is because the combination of $E_{T}^{3}$ is larger than $E_{T}^{2}$ when the number of gathering events is large. The trend of the results obtained using ADDC is similar to that under the physical clock. However, the values obtained with ADDC are larger than those obtained under the physical clock, since we only specify the “happen before” constraint, whereas detection under the physical clock needs a gap of 500 s. When the gap is decreased, the difference between the two approaches becomes progressively smaller.

We then change the duration of a promotion from 500 to 1100 s, and the result is shown in Figure 7. The number of $E_{T}^{2}$ and $E_{T}^{3}$ detected by ADDC is quite similar in this case, since we only use the start time of each event to determine the “happen before” relation; such a relation is not sensitive to the duration of promotion activities. In contrast, the results obtained under the physical clock are more related to the duration of the promotion activity with regard to $E_{T}^{3}$, which is determined by the end time of the first event to the start time of the following event. The results of ADDC are larger than those under the physical clock, since its constraint is less strict.

We subsequently change the affecting distance of marking activity from eight to 22, and the result is shown in Figure 8. It is shown that the increase in distance leads to an increase in the number of $E_{T}^{2}$ and $E_{T}^{3}$. The number of $E_{T}^{3}$ increases faster than that of $E_{T}^{2}$. The result of ADDC is similar to that under the physical clock, but with larger values. The result is consistent with those obtained for different numbers of groups and different durations of activities.

Finally, as shown in Figure 9, we change the interval between two promotions to check the number of event patterns. It can be seen that when this interval increases, the number of event patterns detected by both ADDC and the physical clock decreases. This is because the increased interval reduces the number of gathering events; hence, the number of event patterns decreases. The number of event patterns detected by ADDC is larger than that detected by the physical clock, which is consistent with previous results.

### 5.3. The Comparison under Different Causality Definitions

We then compare the causality relations detected by ADDC and the physical clock based on different definitions. In each simulation, we consider multiple definitions, including $E_{T}^{2}\left(start-start\right)$, $E_{T}^{2}\left(end-start\right)$ and $E_{T}^{2}\left(start-start\;\&\;end-end\right)$. By default, *T* is set to 500 for the physical clock and is not used in ADDC. The detailed descriptions of these definitions are presented in Section 4.2 and Figure 5. The result of the comparison can be seen in Figure 10, Figure 11 and Figure 12.

As shown in Figure 10, the number of event patterns detected under different definitions all increase with the number of groups, but show different features. The number of event patterns under $E_{T}^{2}\left(start-start\right)$ is larger than that under $E_{T}^{2}\left(end-start\right)$. This is because the constraint of $E_{T}^{2}\left(end-start\right)$ is stricter than $E_{T}^{2}\left(start-start\right)$. If an event pattern satisfies $E_{T}^{2}\left(end-start\right)$, it also satisfies $E_{T}^{2}\left(start-start\right)$. However, it does not always hold in the reverseway. Similarly, the number of event patterns under $E_{T}^{2}\left(end-start\right)$ is larger than that under $E_{T}^{2}\left(start-start\;\&\;end-end\right)$. Although the constraint of $E_{T}^{2}\left(end-start\right)$ does not seem to be related to $E_{T}^{2}\left(start-start\;\&\;end-end\right)$, the latter has more constraint sub-expressions; hence, it is generally more strict. According to the figure, the results with the above constraints have a similar value when the number of groups is 75, and the gap between them is increased when the number of groups is increased.

It can also be seen that the event patterns detected by ADDC are larger than those obtained under the physical clock, using the same definition. This is because the specification of *T* increases the strictness of the detection under the physical clock, whereas the detection by ADDC does not have such a restriction.

We then fix the number of groups at 150 and change Tto check the result. The result can be seen in Figure 11. The number of event patterns detected by ADDC under all of the definitions is constant. This is in line with the fact that ADDC is based on message passing rather than on the physical clock; hence, the results are not related to *T*. The number of of event patterns detected under the physical clock increases when Tincreases. This is because a larger value of T relaxes the constraints of a causality relation. When T increases, the gap between the values obtained by ADDC and the physical clock is reduced, because the constraint is relaxed. The difference between the event patterns detected by $E_{T}^{2}\left(start-start\right)$ and those detected by $E_{T}^{2}\left(end-start\right)$ is stable under ADDC or physical time. This also holds for other definitions of causality relations.

After that, we further check the result for different affecting distances. The result can be seen in Figure 12. According to the figure, the number of event patterns detected by both ADDC and under the physical clock increases when the affecting distance increases. This is because a larger affecting distance enables more consumers to receive promotion information and to engage in more gathering events. The number of event patterns detected by ADDC is larger than that under the physical clock using the same definition of the causality relation. The differences among definitions of causality relations are similar to those of previous results. The number of event patterns based on $E_{T}^{2}\left(start-start\right)$ has the largest value, whereas that based on $E_{T}^{2}\left(start-start\;\&\;end-end\right)$ has the smallest value.

### 5.4. Common Event Patterns and the Number of Messages Passed

We further investigate the number of common event patterns detected by ADDC and the physical clock. The result is shown in Figure 13 and Figure 14. As shown in Figure 13, the number of event patterns common to the two approaches increases when the number of groups increases. There are several reasons for the difference. First, the gap between two events under the physical time cannot be described accurately using message passing; hence, some event patterns can be detected by ADDC, but not under the physical clock. Second, the delay resulting from message passing prevents some event patterns from being detected by ADDC, but not under the physical clock.

Figure 13 also shows the maximum number of messages when detecting $E_{T}^{2}$, which increases at a constant rate when there are fewer than 175 groups, but stabilizes beyond 175. This shows that in the latter case, the number of groups is sufficiently large and has little effect on the detection.

Figure 14 shows the common events and the maximum number of messages between two events when adjusting the affecting distance. The results are similar to those in Figure 13. When the affecting distance increases, the number of common events increases, and the maximum number of messages between two events also increases. This is consistent with the uncertainty of asynchronous communications.

## 6. Related Work

To the best of our knowledge, we are the first to investigate group characteristics in relation to consumption and to use the term group consumption. However, related research pertaining to mobile commerce (or mobile purchases) in different fields, including business management, marketing, psychology and computer science, has been reported.

The work from fields other than computer science focuses on finding out the relations between marketing strategies and purchases based on mobile devices. In [14], Molitor *et al*. found that geographical distance is an important factor affecting the behavior of consumers when they use mobile devices. After receiving coupons via mobile devices in a pull way, consumers are more likely to choose a coupon from neighboring shops. In [15], Luo *et al*. investigated how both the promotion time and geographical distance affect the purchase. They found that: for consumers located near the promotion venue, it is more effective to send the promotion information on the promotion day rather than in advance, whereas for consumers far from the promotion venue, it is more effective to send the promotion information on the day before the promotion rather than on the same day or two days in advance. In [16], Andrews *et al*. investigated how crowdedness in a space affects advertising via mobile devices. They found that a crowded environment (e.g., the subway during rush hours) led to more successful advertisements, which they explained in terms of the constraint of physical places increasing people’s interest in advertisements. In [17], Molitor *et al*. investigated how the weather affects people’s tendency to accept coupons sent from mobile applications. In [18], Harmon *et al*. studied the privacy issues of marketing activities based on mobile devices. None of these researchers considered how the relations in a group affect the purchases. In [19], Fisher pointed out that individuals may perform group-derived behavior that is mandated because they are part of a social role the individual has accepted and not necessarily because they are intrinsically satisfying. For example, as one of the fans of a sports team, they may buy gate ticketsand team-licensed products. The behavior can be affected by the attractiveness of the group and the similarity between group members. However, this research is not specific to mobile group consumption.

The field of computer science has seen the publication of many papers concerning mining the rules of mobile users and predicting a user’s location or path. Tseng and Tsui mined the associated rules between the movement of people and the services they requested in different locations [20]. They took into account the multilevel properties of locations and services. In [21], Tseng and Lin proposed an approach named SMAP-Mine to discover patterns in users’ mobile access that contain both movement and service requests. SMAP-Tree was subsequently used to organize the data efficiently. Yun and Chen mined sequential patterns in a mobile commerce environment [22]. They utilized the relationship between the movement and the purchases made by users to detect patterns. The evaluation showed that the results produced by using this approach were more accurate compared to when using only movement or purchase data. In [23], Hadjiefthymiades and Merakos used a linear reward-penalty reinforcement learning method for location prediction. This approach needs many possible transitions of the user’s mobility patterns for training before prediction. In [24], Akoush and Sameh used a hybrid Bayesian neural network model for prediction. Anagnostopoulos and Hadjiefthymiades further eliminated the noise (typically manifested as small-random deviations from previously-seen patterns) from movement patterns using the optimal stopping theory and predicted the user’s k-step-ahead location [25]. The approach kept the knowledge base with the lowest possible total spatial variance. The approach used a delay-tolerant decision-making mechanism, such that it did not require a training phase, but can incrementally refine the knowledge base. In [26], Jeung *et al*. proposed the *trajectory pattern tree* to organize a large number of discovered trajectory patterns. The tree was used to index the trajectory patterns to enable predictive queries to be answered efficiently. A hybrid model based on the trajectory patterns and mathematical motion function was built to rapidly predict the movement of people in the near and distant future. In [5], Lu *et al*. collected information about users’ movements and purchase transactions via mobile commerce for mining and prediction purposes. They proposed a framework named Mobile Commerce Explorer (MCE), which improved upon previous results by (1) considering the similarity between shops and between items in pattern mining and prediction; (2) mining the movement patterns of individuals and (3) predicting the behavior of consumers in terms of both their movements and purchases. Although these results are useful in terms of data analysis processes, they do not consider the relations in a group.

Some researchers also directly investigated ways to promote mobile consumption. In [4], Namiot *et al*. localized consumers based on their wireless network access information and then provided them with customized commercial information (e.g., deals, discounts and coupons). Yang *et al*. collected websites browsed by users and their locations and then recommended new retailers to them [6]. Kanda *et al*. collected users’ real-time trajectory based on a laser rangefinder and particle filter and then predicted the time and venue in which a consumer probably performed consumption [27]. After that, a robot was dispatched to provide various services. In [28], Guo *et al*. investigated how to use RFID technology to promote mobile commerce. In [29], Komninos *et al*. built the system “me-Commerce” and pointed out that various types of context information are important for mobile consumption. These researchers did not consider the scenarios of group consumption. In [30], Zhu *et al*. addressed the problem of discovering user groups based on their similar movement behavior. They proposed a framework to identify such groups by firstly constructing trajectory profiles of users, then deriving similarity between trajectory profiles and, finally, discovering the groups. Anagnostopoulos *et al*. proposed an incremental way to form mobile user groups and to investigate how to validate the groups using an optimally-scheduled and adaptive mechanism [31]. Although these results are useful for mobile group consumption, more investigation is needed.

Moreover, all of this work is based on a centralized server and synchronized clocks, which are prone to a performance bottleneck, single-point failure, synchronization overhead, *etc*. and are sometimes even infeasible because of hardware constraints or privacy concerns. In [32], Anagnostopoulos described a distributed time-optimized forwarding way to collect contextual information from a source node to a consumer node in mobile sensor networks. This work is based on synchronized clocks.

The field of distributed computing has produced work relating to asynchronous distributed data collection. The uncertainty of asynchronous distributed computing led to the introduction of *definitely* and *possibly* modalities in [33] for detecting events. The lattice was invented as a tool for detecting generic events, named *relational predicates* [33,34]. A special class of predicates, *conjunctive predicates*, specified by a conjunctive expression of local states, was also investigated [9]. In [13], Zhu *et al*. used occurrence probability to refine the *possibly* modality to provide more detailed information and can support the detection of multiple occurrences of events. In this paper, we follow the basic idea of asynchronous distributed computing and adjust it for mobile group consumption.

## 7. Conclusions

In this paper, we propose an asynchronous distributed approach to collect data relating to mobile group consumption. Considering that many scenarios involve a great number of consumers who constantly move, our approach is scalable and efficient, because it neither needs a central server nor a synchronized clock. We first built a system model based on asynchronous distributed communications. Then, we proposed a three-layer solution framework. Three kinds of processing, including the processing in a local sub-region, in consecutive sub-regions and in several sub-regions, are considered. Subsequently, we demonstrate how to detect the causality relation among two/three gathering events happening in the system based on message exchange. Three definitions of the causality relation are supported. We conduct extensive simulations to validate the proposed approach. The results show that our approach is effective.

## Figures and Tables

**Table 1:** Notations in the algorithms.

Variable Name	Description
*shopID*	ID of a shop
PROMOTION(shopID)	promotion information broadcast by *shopID*
*accepted*	the variable denoting whether a consumer accepts a promotion
*location*	current location of a consumer
*initiator*	the first shop sending the message
*sender*	the previous shop sending the message
*eventID*	ID of an event
*depth*	the depth of *sender* in the routing tree
*recClock*	the vector clock attached to a message
*clock*	the vector clock maintained by a consumer or a shop
*cardinality*	the number of consumers in a shop
*persons*	a collection recording detailed consumer IDs in a shop
*personID*	ID of a consumer
*increaseID*	a function to generate the ID of a new event
*gatherThreshold*	the threshold of gathered consumers to denote the occurrence of a gathering event
*detFlag*	the type of causality relation to be detected
*eventList*	a list of events received by the current shop
*oriStart*[*eventID*]	the initiator of *eventID* after it starts, as the current shop knows
*oriEnd*[*eventID*]	the initiator of *eventID* after it ends, as the current shop knows
*SS*[*preID*]	a flag recording the detection result of the first part of SSEE ($E^{2}\left(start-start\;\&\;end-end\right)$)
*parent*	the parent of the current shop in the routing tree
*children*	the children of the current shop in the routing tree
*depth*[*eventID*]	the parent of the current shop in the routing tree regarding *eventID*
MSG_NTER(*A*)	a message denoting that *A* enters the current shop
MSG_OUT(*A*)	a message denoting that *A* exits the current shop
START	a message denoting the start of a gathering event
END	a message denoting the end of a gathering event
SUCCESS	a message denoting a successful detection of a causality relation
REVERSE	a message to notify the reverse of the routing tree
E	the specification of an event that needs to be detected
*coordinator*[E]	denotes whether the current node is the coordinator of E

## Abbreviations

The following abbreviations are used in this manuscript:

GCS: Consumption Simulation System
ADDC: Asynchronous Distributed Data Collection Approach
